# The sharing economy in the hospitality sector: The role of social interaction, social presence, and reciprocity in eliciting satisfaction and continuance behavior

**DOI:** 10.1057/s41599-022-01379-y

**Published:** 2022-10-11

**Authors:** Linda Heejung Lho, Wei Quan, Jongsik Yu, Heesup Han

**Affiliations:** 1 Sejong University, Seoul, Republic of Korea; 2 Cheongju University, Cheongju, Republic of Korea

**Keywords:** Business and management, Psychology

## Abstract

The sharing economy is still transforming the hospitality industry at an exponential speed. The idea of acquiring goods and services through a collaborative approach is becoming a significant part of the world’s overall economy. Many use platforms and social media channels to make purchase decisions while communicating with others. This study, therefore, investigates the socialization factors and values of the sharing economy as driving forces of the rapid growth of the hospitality businesses related to sharing economy. The study also assesses their influence on satisfaction and continuance behavior and explores the role of reciprocity and perceived risk by applying the value-based adoption theory. Using mixed methods, the present research identifies the crucial attributes and validates the proposed theoretical framework. Our findings provide valuable insights for hospitality businesses participating in the sharing economy.

## Introduction

The “sharing economy” is a peer-to-peer transaction that may or may not involve monetary exchange. It can be termed as an “activity of sharing, lending, and acquiring of goods” (Hamari et al., 2016) without involving interference from a third party. The sharing economy seems like an urban phenomenon since the 2000s. Yet, the “sharing economy initiatives have been around for a long time, such as a time-share ownership of vacation properties, ride-sharing arrangements, and the like” (Hira and Riley, 2017). An act of sharing is not only the fundamental form of commercial activity in human society for many years and it is also detectable in animals’ biological behavior (Martin, 2016). Actions such as sharing food, clothes, toys, etc., between siblings and lending unutilized goods to people in need have always been with us in our society. Due to the emphasis on the fundamental form of economic distribution in our society, several studies on the sharing economy have investigated customers’ social behaviors in the sharing economy and their effects on customer satisfaction in using the hospitality businesses participating in the sharing economy (Priporas et al., 2017). A high level of social interaction within the sharing economy was revealed to affect customers’ perceived service quality (Pitt et al., 2020), which is likely to influence customer satisfaction. Moreover, several studies have investigated the motivation to participate in the sharing economy according to self-determination theory (Li and Wen, 2019).

Nevertheless, the research gap still exists in the hospitality literature in the context of the sharing economy. First, while the previous studies confirm the factors of the sharing economy in different contexts (e.g., Li and Wen, 2019; Möhlmann, 2015), research on what attributes influence customers’ intention to use the hospitality businesses participating in the sharing economy is not yet clearly identified. Second, regarding the sharing economy, a number of existing research prevalently apply social exchange theory (SET) which emphasizes the intention to choose a relationship that is most beneficial, (Priporas et al., 2017), or self-determination theory which highlights the personal decision-making process from intrinsic and extrinsic motivation (Li and Wen, 2019). The attributes of social factors in relation to the perceived values of the sharing economy remain unearthed and very limited studies have identified the effect of cost (Kim et al., 2019). However, social cognitive theory by Zhu et al. (2017) states that social values along with emotional and functional values are fundamental antecedents to perceived value (Zhu et al., 2017). This study thus applies both Zhu et al. (2017) to analyze the influence of perceived value (social, emotional, and functional) and Kim et al. (2007)’s value-based adoption theory to analyze factors of the hospitality businesses participating in the sharing economy and the influence of risk. Likewise, this study identifies the moderating role of perceived risk and reciprocity of the sharing economy to sustain the qualitative growth of the hospitality businesses participating in the sharing economy. For this reason, this study was designed to fill the research gaps by identifying the attributes of the hospitality businesses participating in the sharing economy which affect customer satisfaction and continuance intention. This study thus explicitly aims to (1) identify the major attributes of the hospitality businesses participating in the sharing economy; (2) explore the effects of extrinsic and intrinsic values on customer satisfaction and continuance intention, and (3) discover the moderating role of perceived risk and reciprocity in the relationships among customer satisfaction and continuance intentions.

As the sharing economy will continue its phenomenal growth and more businesses participating in sharing economy will continue to emerge, the necessity to examine the influential attributes of the hospitality businesses exists. This study hence discovers the attributes of the hospitality business participating in the sharing economy through qualitative and quantitative methods and analyzes the correlation between the proposed attributes through qualitative methods. Moreover, this study explores the relationships between customer social interaction, extrinsic and intrinsic values of hospitality businesses, customer satisfaction, and continuance intention. The study also presents a new insight by discovering the moderating role of perceived risk and reciprocity in the relationship between customer satisfaction and continuance intentions. A detailed literature review of the study variables is presented below. This study adopts qualitative person-to-person interviews and an online survey method. The data was then analyzed through factor analyses, structural equation modeling, and invariance tests.

## Literature review

### Sharing economy

The sharing economy, also known as collaborative consumption (Botsman and Rogers, 2010), a collaborative economy (Gyimo´thy and Dredge, 2017), and access-based consumption (Botsman, 2014), is an “activity of sharing, lending, and acquiring of goods and/or services” (Hamari et al., 2016) through a peer-to-peer transaction. This study adopts the definition of the sharing economy mainly but takes collaboration amongst individuals into consideration.

Of the four types of the sharing economy business platforms: peer-to-peer, business-to-business, business-to-peer, and crowd (Curtis and Mont, 2020), the two most popular types are peer-to-peer and business-to-peer (Li et al., 2020). Examples of peer-to-peer businesses are Uber and Airbnb, which allow transactions between customers, and an example of a business-to-peer platform is Spacious, a coworking platform in NYC (Curtis and Mont, 2020). Focusing on the three categories of hospitality businesses participating in the sharing economy (peer-to-peer accommodations, coworking spaces, and shared kitchens), this research provides a general insight into customers’ behavioral intentions in the sharing economy in the context of the hospitality industry.

**Fig. 1:**
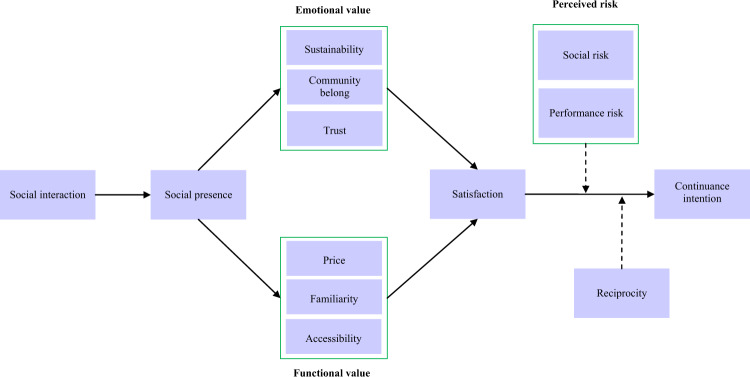
The proposed conceptual model shows the study’s conceptual model. The model examines the relationship between social presence, three emotional and three functional factors, satisfaction and continuance intention. The moderators are perceived risk and reciprocity.

As many hospitality business platforms are multi-sided platforms, which “promote social cohesion” and create a sense of community (Curtis and Mont, 2020), this research examines the socio-economic system of the sharing economy to observe the social interaction between customers as an antecedent to the intention to participate in the sharing economy. The research also explores perceived values and risks of the sharing economy and measures their effects on customers’ continuance intention (Fig. 1).

### Social cognitive theory

According to Bandura, social cognitive theory classifies human behavior in relation to triadic, reciprocal, and dynamic interaction (Bandura, 2001; Wang et al., 2019). Zhu et al. (2017) described SCT as a theory explaining human behavior is a value-driven behavior. Referring to Zhu et al.’s view of SCT, the research developed a study model to analyze the social value, functional value, and emotional value as the critical components of the perceived value influencing the behavior of the sharing economy participants.

### Value-based adoption theory

Value-based adoption theory (VAM) proposed by Kim et al. (2007), claims that the technology acceptance model (TAM) is limited in explaining acceptance of new technology from customers’ perspective and measuring cost/sacrifice (Kim et al., 2019). TAM is one of the most widely applied theories along with the unified theory of acceptance and use of technology (UTAUT) (Liu et al., 2020) when analyzing the adoption of technology. However, because TAM, which considers perceived usefulness and ease of use (Huang et al., 2019; Davis, 1989), and UTAUT, which is an extended version of TAM (Assaker, 2019) fail to investigate the beneficial attributes (intrinsic and extrinsic values) and cost (perceived risks), VAM is applied to analyze the perceived values and costs as the antecedents to satisfaction and continuance intention.

### Social interaction within sharing economy

Social interaction is defined as an “exchange between two or more individuals”, which is a fundamental component of a society or a social network (Lumen Learning, 2020). The sharing economy, which is known as a socio-economic ecosystem, requires individuals to socially interact and collaborate to make a purchase or an exchange. Social interaction in the sharing economy is thus defined as “any actions an individual engages in that affect other consumers’ valuations or decisions regarding a product or service” (Godes et al., 2005; Wang and Yu, 2017).

The major hospitality businesses participating in the sharing economy are Airbnb, WeWork, and Garage Kitchen (e.g., Cao et al., 2022). Although Airbnb has been around for over 10 years, it is still “the most prominent example of the sharing economy in hospitality and is becoming an important player in the hospitality sector” (Priporas et al., 2017). Not just limited to tourism, the real estate industry is also experiencing shifts due to the booming coworking spaces and shared kitchen businesses. Although the two businesses use less of their platforms, they still initiate social interaction offline amongst customers and potential customers and amongst customers and themselves. Customers of peer-to-peer accommodation, coworking spaces, and shared kitchens exchange information such as pre-purchase decisions in both online and offline communities. Thus, these platform businesses are considered social commerce which is a mixture of shopping and socialization (Wang and Yu, 2017). “The development of social technologies reintroduces the social side into the online purchasing process, making online purchasing a more social experience” (Lu et al., 2016). Also, social commerce is an exchange-related activity that occurs in one’s social network in an internet-mediated social community to make “recognition, pre-purchase, purchase, and post-purchase” decisions of a product and/or service (Wang and Yu, 2017; Yadav et al., 2013).

Many previous studies focused on the observational learning aspect of social interaction to understand customer behaviors. However, other social influences within the social interaction such as social presence have not been investigated as much. According to Zhang et al., understanding social support, social presence, and flow will increase social commerce intention for customers using technological features (Zhang et al., 2014). Of the three factors, social presence is known to be the most considerable factor within social interaction as customers receive more information and social signals to make their purchase decisions from the social environment (Lu et al., 2016). “Social presence is built upon signals transmitted in a communication medium” (Lu et al., 2016). Thus, social interaction within the hospitality businesses participating in the sharing economy’s platform will also develop a social presence, which will be influential to the customer’s purchasing decision. Consequently, social interaction and social presence are directly related (Horzum, 2015). This research hence hypothesizes the following hypothesis:

Hypothesis 1: Social interaction in the hospitality businesses participating in the sharing economy’s platform is positively related to the social presence within the hospitality business participating in the sharing economy.

### Social presence within sharing economy

Social presence, which is also known as presence, is “the salience of others in a mediated communication” (Short et al., 1976), meaning that it is an intrinsic element of the communication medium (Lu et al., 2016). Social presence can also be viewed as intimacy and closeness in a relationship between two or more individuals (Lu et al., 2016; Short et al., 1976). Thus, from a psychological viewpoint, social presence in e-commerce implies the warmth, personal, and sociable aspects of the website (Cyr et al., 2007).

Face-to-face communication brings high social presence than paper-based communication (Gefen and Straub, 2004). However, this does not imply that platforms involving social interaction can only embed low social presence (Gefen and Straub, 2004). Platforms can encourage high social presence simply by adding pictures and videos. The hospitality businesses participating in the sharing economy often make use of social commerce to provide their customers with real customers feedbacks, giving social proof to their customers when making their purchase decisions. Encouraging social interaction online through personalized chat and review systems, these hospitality businesses participating in the sharing economy generally arouse a high social presence.

High social presence from consumer-oriented platforms reduces ambiguity, increases trust, and encourages users to purchase with lower levels of consumer dissonance (Cyr et al., 2007). Therefore, the platforms of the hospitality businesses are likely to involve much social interaction between the business and the customers to reduce any uncertainties that customers may have about the businesses’ services. “Communication is a necessary ingredient of constructive interaction” and increased electronic communication may encourage higher perceived social presence, which is likely to increase trust (Gefen and Staub, 2004).

Although extant studies have revealed a positive relationship between social presence and trust (Al-Ansi et al., 2019), the relationships between social presence and other emotional and functional values remain untouched. Since high social presence is usually present with the richness of information on the hospitality businesses’ platforms it is likely to affect the hospitality businesses’ perceived values. Hence, this research proposes the following hypothesis:

Hypothesis 2: Social presence in the hospitality businesses participating in the sharing economy’s platform is significantly related to a sense of sustainability of the services.

Hypothesis 3: Social presence in the hospitality businesses participating in the sharing economy’s platform is significantly related to a sense of community belonging to the services.

Hypothesis 4: Social presence in the hospitality businesses participating in the sharing economy’s platform is significantly related to trust in the services.

Hypothesis 5: Social presence in the hospitality businesses participating in the sharing economy’s platform is significantly related to the price of the services.

Hypothesis 6: Social presence in the hospitality businesses participating in the sharing economy’s platform is significantly related to the accessibility of the services.

Hypothesis 7: Social presence in the hospitality businesses participating in the sharing economy’s platform is significantly related to familiarity with the services.

### Trust

Trust is generally defined as the “willingness of a party to be vulnerable to the actions of another party based on the expectation that the other will perform a particular action important to the trustor, irrespective of the ability to monitor or control that other party” (Han et al., 2019; Hong and Cho, 2011; Mayer et al., 1995). In consumer marketing, trust is defined as a “consumer’s perceived reliability on the brand, products, or services of a merchant” (Hong and Cho, 2011; Flavian et al., 2006). This research defines trust mostly in the characteristic of consumer marketing and extends the defined definition of trust by taking the trust of intermediaries and sellers into consideration (Al-Ansi and Han, 2019; Yu et al., 2022).

Trust is a vital component of the act of sharing (Song et al., 2019; Hawlitschek et al., 2016; Belk, 2014). For one to share a product with a friend, the owner of the product must have trust in the friend. Vice versa in some cases, service users/customers need trust in the seller/service provider to purchase the service. To better assure and connect both parties, the role of the intermediaries is very important in an online market as the customers find making online purchases from an unfamiliar merchant very uncomfortable (Hong and Cho, 2011). An e-commerce platform thus acts as the intermediary between the seller and consumers “to apply guarantees, regulations, safety nets, or other structure” (Hong and Cho, 2011).

As previously mentioned, trust in e-commerce can be established with a high social presence (Cyr et al., 2007). Plenty of information and abundant social interactions within the platforms of hospitality businesses will increase trust. This increased trust in the hospitality businesses participating in the sharing economy’s service is likely to lead to increased satisfaction. Kim et al. (2008) revealed that consumer post-purchase satisfaction is significantly related to e-commerce trust. Therefore, this research proposes the following hypothesis:

Hypothesis 8: Trust in the hospitality businesses participating in the sharing economy is significantly related to satisfaction.

### Community belonging

A few researchers defined community belonging as a neighborhood attachment creating a social bonding and behavior rootedness (McMillian and Chavis, 1986; Riger and Lavrakas, 1981). However, some started to view community belonging as a relational aspect. Community belonging was seen as the “quality of character of human relationship, without reference to location” (McMillian and Chavis, 1986). It is created around “interest and skills more than around locality” (McMillian and Chavis, 1986; Durkheim, 1964). And thus, people tend to enjoy being in a community, which is very likely to be related to a shared emotional connection.

The role of collective co-production and a community in the sharing economy has been highlighted by numerous researchers (Möhlmann, 2015). In the sharing economy, people interact with each other creating a community, where they rent, lend, and exchange goods, services, and information. The feeling of community belonging can be observed not only in the sharing economy but also just in the general economy. Joining the brand communities, for example, the customers make purchases of the brands’ products correspondingly to the shared information and eventually become loyal customers of those brands. Thus, many researchers stress that a sense of community is very closely related to consumer behavior.

A sense of community belonging can be easily observed in peer-to-peer accommodations, coworking spaces, and shared kitchens. Peer-to-peer accommodations allow the customers to interfere with the locals and other peer customers, creating a community amongst themselves both online and offline. Consequently, peer-to-peer accommodation customers tend to have a higher chance of creating special experiences than those of hotels. The coworking spaces provide a shared working environment allowing independent workers to mingle to “create knowledge and benefit” from an association with each other (Spinuzzi et al., 2019). Airbnb, for example, promises its customers to provide “closer interactions with [locals] and to create a sense of place” (Tussyadiah and Peasonen, 2016) while WeWork builds a community that “shapes employees’ experiences and their engagement in jobs” (La Pietra and Rowell, 2019) by encouraging interactions between tenants and employees. The shared kitchen is also very similar to the coworking space as it rents out kitchen spaces for delivery-only restaurants, allowing restauranters to communicate with each other. These interactions between customers create a sense of community belonging, which is likely to affect satisfaction. Thus, a sense of community belonging is known as a major driver of participation in sharing economy (Möhlmann, 2015). Additionally, customers who belong to a community are likely to develop higher levels of general satisfaction (Royo-Vela and Casamassima, 2011). Therefore, the following hypothesis is developed:

Hypothesis 9: Community belonging in the hospitality businesses participating in the sharing economy is significantly related to satisfaction.

### Sustainability

Sustainability is generally described as a subdivision of sustainable development, which satisfies the needs of the current generation without depriving the resources of the next generation (Midgett et al., 2018). Sustainability in collaborative consumption is defined as the amount to which the consumers observe they can save resources and minimize consumption by participating in the sharing economy (Li and Wen, 2019). In the hospitality industry, sustainability is also big. Not just limited to the usage of green products in hotels and resorts, businesses like peer-to-peer accommodations are good examples of major sustainable businesses.

Peer-to-peer accommodations allow owners to utilize their vacant properties and give opportunities for travelers to stay in these properties, creating a resourceful community. The coworking spaces have four constituents to their sustainability aspect: “New Work”, “Resourceful Society”, “Environmental”, and “Incubator” (Oswald and Zhao, 2020). “New Work” highlights the flexible nature of working conditions and sharing of resources, “Resourceful Society” emphasize efficient energy use while taking the social factors into account, “Environmental” stresses the environmental factors of the spaces, and “Incubator” highlights the community formed within the coworking spaces (Oswald and Zhao, 2020), making the coworking spaces sustainable. The customers believe they have contributed to others, society, and nature (Li and Wen, 2019) by making sustainable choices. This belief is likely to bring positive emotions to customers. Consequently, this study hypothesizes the following:

Hypothesis 10: Sustainability in the hospitality businesses participating in the sharing economy is significantly related to satisfaction.

### Price

A price is the number of dollars that buyers must sacrifice to acquire a product or service or is defined as the monetary value (dos Santos Silva et al., 2021). The sharing economy, which is renowned for offering an unutilized product or service at a lower cost, is hence growing very rapidly. The economic value in the sharing economy is the most prevailing factor when discussing the value of collaborative consumption (Barnes and Mattson, 2017). The price in the sharing economy is therefore an influential factor affecting consumers’ purchase decisions.

Since there are many businesses participating in the sharing economy within the hospitality industry, consumers are easily finding affordable options to travel and work. Peer-to-peer accommodations provide affordable options for travelers while coworking spaces and shared kitchens offer flexible lease contracts. One of the key motivations to use peer-to-peer accommodations is the economic benefits (Barnes and Mattson, 2017). As such, many tenants view the coworking spaces economical than renting offices individually. The shared kitchen services are also undertaking a rapid expansion worldwide as they lower the market entry costs for food and beverage startups (Shin, 2019). These economical options of the hospitality businesses participating in the sharing economy thus are likely to positively influence customer satisfaction when using the sharing economy services:

Hypothesis 11: Price/economic benefit of the hospitality businesses participating in the sharing economy is significantly related to satisfaction.

### Familiarity

Familiarity in the sharing economy originates from a close acquaintance with the brands or products (Kuzhady et al., 2020). This familiarity commonly reduces the uncertainty which exists before a purchase. Hence, increasing familiarity with the brands or products will positively influence the customers’ purchase intention (Kuzhady et al., 2020). This tendency is also visible in the hospitality industry. Many previous studies have revealed that travelers tend to choose familiar travel destinations. Destination familiarity affects the travel intentions and the travel decisions of customers (Chi et al., 2020).

The familiarity of the hospitality businesses participating in sharing economy is increasing every year with the sharing economy boom. Accordingly, it is very likely that consumers observe this familiarity as the uncertainty reducer of the sharing economy services. With an increase in the familiarity from abundant information through social interaction between customers, the customers of the sharing economy are likely to appreciate the usefulness of the services. This appreciation of familiarity in the hospitality industry’s sharing economy is likely to lead to consumer satisfaction. This research proposes the following hypothesis:

Hypothesis 12: Familiarity in the sharing economy positively influences satisfaction.

### Accessibility

Accessibility is “the quality of being able to be reached or entered” (Oxford Languages, 2021). Accessibility in the sharing economy is especially important. Easily accessible information on peer-to-peer networks allows customers to freely browse and choose the service or product they are looking for. Many peer-to-peer sharing services are facilitated by online platforms which positively influence collaborative consumption (Möhlmann, 2015). Allowing easy communication among customers is known as the key constituent of collaborative consumption (Möhlmann, 2015; Ostrom, 1990).

As mentioned previously, the major hospitality businesses participating in the sharing economy offer online platform services for customers to communicate with each other. Airbnb, WeWork, Garage Kitchen, etc., all utilize the internet and mobile applications to introduce their services and communicate with customers. Thus, this research views that the accessibility of the business services through internet capabilities adds value to the functional values of the sharing economy positively impacting customer satisfaction:

Hypothesis 13: Accessibility in the sharing economy positively influences satisfaction.

### Satisfaction

The existing studies commonly define satisfaction as an “emotional state following virtual community usage” (Sthapit et al., 2019) or “emotional state from post-purchase feeling” (Williams and Soutar, 2009). However, a uniform definition of consumer satisfaction is the “summary affective response of varying intensity…with a time specific point of determination and duration” toward a certain product or service (Giese and Cote, 2000).

The extant literatures on satisfaction in the hospitality industry are mostly focused on service quality satisfaction, which is the significant constituent of repeat purchases (Tam, 2000). Even though customer satisfaction in the hospitality industry is strongly connected to service quality, the perceived value was found to be the antecedent to consumer behavior (Tam, 2000; Cronin et al., 1997). For this reason, this research investigates the intrinsic and extrinsic values of businesses as the antecedents of customer satisfaction.

Satisfaction in hospitality businesses participating in the sharing economy usually originates from the consumers’ intrinsic and extrinsic motivation (Tussyadiah, 2016). The customers feel satisfied when they find enjoyment during their stays in peer-to-peer accommodations. Staying in peer-to-peer accommodations, customers are likely to find enjoyment in traveling like locals. For the coworking space tenants, they are likely to find enjoyment in sharing information with neighbors. Consumers also tend to feel satisfied when they are reducing environmental harm (Botsman and Rogers, 2010; Gansky, 2010) and when gaining monetary benefits (Tussyadiah, 2016; Botsman and Rogers, 2010).

Not only the perceived values are the constituents to purchase and repurchase intention, but also satisfaction affects continuance intention (Sthapit et al., 2019). Innumerable marketing research studies have examined customer satisfaction as a good indicator of positive customer behavior intention (Tussyadiah, 2016) and customer retention (Mittal and Kamakura, 2001). Hence, this research hypothesizes the following:

Hypothesis 14: Satisfaction positively influences continuance intention.

### Continuance intention

A continuance intention is defined as “the strength of customers’ intention to perform a specified behavior” (Sthapit et al., 2019). A number of extant studies on e-commerce and consumer behavior investigated the continuance intention of the customers because the continuance intention can be interchangeably seen as a repurchase intention on online shopping platforms. Numerous research studies have exhibited a positive correlation between satisfaction and repurchase intention (Arlanda and Suroso, 2018), while the consumers with a positive experience on social networking sites were more likely to have a continuance intention than those with negative experiences (Sthapit et al., 2019; Sibona et al., 2017). Thus, instead of using the term repurchase intention, this study selected the term continuance intention to encompass both the intention to participate in the sharing economy involving any monetary purchases and the intention to repurchase the hospitality business products.

### Performance risk

Performance risks refer to the “anxiety about the quality of rental service products” (Lee et al., 2021). As mentioned, many hospitality businesses participating in the sharing economy will operate online platforms to initiate sales of their services. Even though businesses utilizing internet services allow many consumers a convenient consumption of the services, there exists a great uncertainty about the physical products or services. In many cases, the description of the products shown in virtual retail fails to ensure their quality (Marceda Bach et al., 2020). For example, Airbnb rooms shown on the platform may seem very clean and new, but they may be dirty and old in reality. Hence, it is very important to take these perceived performance risks into consideration when studying the consumer behavior of the people participating in sharing economy.

### Social risk

Social risks are the risks that may arise from a lack of “self-identity and social stigma for not properly presenting one’s social status” (Lee et al., 2021). Purchasing rental items may increase the chance of being mistreated or criticized by friends and families (Lee et al., 2021). The same could apply to the users of the hospitality businesses participating in the sharing economy. Consumers using Couchsurfing are likely to face social risk, as they will not want to represent their social status through their stays on Couchsurfing. As social networks become popular, there is an increase in the number of people who like to express their social status online with luxurious content. For that reason, as the sharing economy has an image of economic benefits, there exists a chance of social risks.

Hypothesis 15: Performance risk negatively influences the positive correlation between satisfaction and continuance intention.

Hypothesis 16: Social risk negatively influences the positive correlation between satisfaction and continuance intention.

### Reciprocity

Reciprocity in the business is defined as a reward from customers in return for sellers' or service providers’ exceptional services (Zulkifili and Yazid, 2020). According to Nava et al. (2019), cooperative interaction such as reciprocity naturally develops at a very early age and our society is organized around this interaction. Thus, in the business world, positive reciprocity usually brings a positive influence on businesses’ growth. Vice versa, negative reciprocity is known to lead customers to negative behavioral actions (Zulkifli and Yazid, 2020). Considering the theory of reciprocity, this research hypothesized the following hypotheses:

Hypothesis 17: Reciprocity positively influences the correlation between satisfaction and continuance intention.

## Methods

### Measurement development

The relevant measurement items were carefully chosen from extant literature and justified from a person-to-person interview to develop a self-administered survey. A thorough literature review of attributes of the sharing economy was performed before conducting person-to-person interviews to understand the sharing economy. Then, six interviews with the hospitality business users were done in depth to identify potential common attributes of the three hospitality business sectors participating in the sharing economy. Two practitioners from the three hospitality businesses who have been using the service at least twice or two months long were interviewed. The interviewees selected were all Korean and in their 30s. The interview began with an introduction outlining the purpose of this study to find common attributes of the hospitality businesses in the sharing economy. Open-ended questions were asked during the interview for 20min to guide participants to brainstorm the attributes on a large scale. The interview consisted following questions: (1) What kind of emotional and functional values are important in hospitality business services? (2) How would you describe collaboration using hospitality business services? (3) What is the most important attribute within an interaction in hospitality business services? Interviewees freely shared their perceptions and stories about the experiences. From the interviews, several keywords which stood out were coded and compared with the attributes selected from the extant literature. An example of a coded keyword from the interviews was “accessibility”, which was coded from responses that the business services are quick and easy to find. Similarly, several interviewees mentioned that socialization within hospitality services arouses an emotional attachment. This was coded as “community belonging”. The interviewees also mentioned the economic benefits of the hospitality business services, which were coded “price”. Consequently, these interviews further justified the selected attributes of the sharing economy from the extant literature. The measurement items for socialization were adopted from (Tussyadiah, 2016) and social presence items were from (Gefen and Straub, 2004). Three statements to measure trust were from (Hawlitschek et al., 2016). For community belonging, three items were adopted (Henning-Thurau et al., 2007). The measurement items by Hamari et al. (2016) were implemented for sustainability. Four statements for the price were from (Tussyadiah, 2016). Familiarity measurement items have been employed (Bhattacherjee, 2002). Risk measurements were from Pavlou and Gefen (2004)’s research. Satisfaction measurement items were adopted from Lamberton and Rose (2012), Möhlmann (2015), and Tussyadiah (2016) while continuance intention measurement items were from Nicolau and McKnight (2006). A 7-point Likert type scale (1 = strongly disagree, 7 = strongly agree) was employed for all the items except for the demographic data (e.g., gender, age, income level).

### Data collection and analysis

The survey was carried out in Korea by an online research company to implement a random sampling method. Before beginning the survey, a short introduction was provided to share the study’s objectives with consent to participate. A filter question “Have you ever used either of the hospitality businesses services?” were asked in the beginning to filter the participants who have used the following services in the past two years, considering the Covid-19 era. To ensure the usage, they were also asked which of the services they have used and when was the last time using the sharing economy services. Additionally, the survey explained that participants’ personal information would be kept confidential. 418 valid samples out of 430 were collected during the 7-day survey period, 12 responses were eliminated due to missing data for empirical analysis.

Demographic analysis was performed to verify the sample characteristics. Among 418 respondents, 49% were men and 51% were women. The age groups were distributed quite evenly. 25.1% were in their 20s, 25.1% were in their 30s, 25.3% and 24.4% were 50s or more. The education level and income level of the respondents were slightly higher than the nation’s average. 73% of the respondents were university degree holders while 11.7% were high school graduates, 10% were from graduate school or higher, and 5.3% were professional college graduates. 33.3% of the respondent's annual income fall between $30,000 and $49,000, 28.9% had income below $30,000, 20.1% between $50,000 and $69,000, and 10.3% between $70,000 and $89,000, and 7.4% over $90,000 when converted to US dollars. Also, the majority (55.7%) of the total respondents answered that they have used the hospitality businesses participating in the sharing economy’s services within 6 months.

For the quantitative approach, exploratory factor analysis (EFA) and correlation analysis using SPSS 20 confirmed the attributes of the hospitality businesses participating in the sharing economy services. Then, confirmatory factor analysis (CFA) and structural equation modeling (SEM) was conducted using AMOS 20 to examine the proposed hypotheses and the study model. To explore the moderating effect of perceived risks and reciprocity, multi-group analyses were performed.

## Results

### Qualitative procedure

The results of in-depth person-to-person interviews with open-ended questions showed similarities in the responses. Accessibility was mentioned in all three hospitality business users. Examples of the interviewees’ opinions are as follows:

…It was very easy for me to access this kitchen. I have chosen this place because the transportation from my house is very convenient, and I was able to sign the contract right away after a quick tour. (shared kitchen tenant, Q1)

Socialization and community belonging were also found to be important. Examples of the interviewees’ responses are as follows:

… I find talking to the neighbor very helpful. We often share information that are helpful to our startup businesses. (coworking space tenant, Q1)

…I cannot forget the hospitality of my Airbnb host. She not only introduced phenomenal restaurants nearby but also offered me to join a dinner with her friends the night I arrived alone in Jeju. (peer-to-peer accommodation customer, Q1)

Moreover, interviewees observed that familiarity and trust in the business services are important factors when choosing to use the shared economy services. For this reason, the interviewees’ responses generally mirrored the attributes selected from the extant literature.

### Confirmatory factor analysis

After identifying the valid attributes for the hospitality business participating in the sharing economy services through an EFA analysis, a CFA analysis was conducted. Table 1 portrays the results of CFA including descriptive analysis, correlations, validity, and reliability. The goodness-of-fit statistic of the measurement model (*x*^2^ = 2704.316, df = 1084, *p* < 0.001, *x*^2^/df = 2.495, IFI = 0.885, TLI = 0.869, CFI = 0.884, RMSEA = 0.060) showed a good model fit, comparing to the threshold (Hair et al., 2014). The composite reliability (CR) values of all measurement variables ranged from 0.590 to 0.911. The majority of measurement items surpassing the minimum level expected of CR is 0.70 (Hair et al., 2014). Then, to examine the convergent validity, the average variance extracted (AVE) values were analyzed. The AVE value, computed from factor loadings of social interaction, sustainability, community belonging, trust, price, familiarity, accessibility, satisfaction, reciprocity, and continuance intention were all over 0.5, which is the minimum recommended amount (Fornell and Larcker, 1981).

**Table 1:** The measurement model evaluation results.

	(a)	(b)	(c)	(d)	(e)	(f)	(g)	(h)	(i)	(j)	(k)	(l)	(m)
(a) Social interaction	0.763												
(b) Social presence	0.858**	0.700											
(c) Sustainability	0.702**	0.680**	0.879										
(d) Community belonging	0.756**	0.769**	0.561**	0.793									
(e) Trust	0.744**	0.714**	0.698**	0.674**	0.767								
(f) Price	0.546**	0.443**	0.382**	0.608**	0.468**	0.809							
(g) Familiarity	0.580**	0.487**	0.455**	0.588**	0.585**	0.622**	0.823						
(h) Accessibility	0.520**	0.477**	0.395**	0.562**	0.563**	0.587**	0.831**	0.820					
(i) Satisfaction	0.636**	0.578**	0.476**	0.624**	0.688**	0.640**	0.718**	0.754**	0.837				
(j) Performance risk	0.530**	0.596**	0.452**	0.477**	0.528**	0.382**	0.434**	0.282**	0.373**	0.570			
(k) Social risk	0.685**	0.757**	0.662**	0.641**	0.781**	0.535**	0.597**	0.573**	0.693**	0.721**	0.718		
(l) Reciprocity	0.432**	0.298**	0.217**	0.402**	0.256**	0.483**	0.385**	0.301**	0.297**	0.837**	0.443**	0.777	
(m) Continuance intention	0.664**	0.570**	0.530**	0.695**	0.708**	0.527**	0.691**	0.727**	0.873**	0.334**	0.710**	0.287**	0.817
Mean	4.75	4.52	4.56	4.89	4.90	4.69	5.05	4.83	4.88	4.52	4.83	4.59	4.26
SD	0.949	0.860	1.206	0.934	0.949	0.983	0.945	0.971	0.991	1.005	0.949	0.854	0.883
CR	0.848	0.657	0.911	0.836	0.810	0.850	0.893	0.911	0.875	0.590	0.761	0.752	0.857
AVE	0.582	0.490	0.773	0.629	0.588	0.655	0.677	0.672	0.701	0.392	0.515	0.603	0.667

Goodness-of-fit statistics for the measurement model: *χ*^2^ = 2704.316, df = 1084, **p < 0.01, *χ*^2^/df = 2.495, RMSEA = 0.060, CFI = 0.884, IFI = 0.885, TLI = 0.869.

### Structural equation modeling

SEM was performed to test the hypotheses. The goodness-of-fit statistics for structural model was acceptable (*x*^2^ = 1356.893, df = 608, *p* < 0.001, *x*^2^/df = 2.232, IFI = 0.928, TLI = 0.921, CFI = 0.928, RMSEA = 0.054). The proposed hypotheses of this research were mostly supported. Hypothesis 1, social interaction is significant to social presence, was supported (H1 *β* = 0.904, *p* < 0.001). According to the results, social presence showed significance toward sustainability, community belonging, and trust which supported hypothesis 2, 3, and 4 (H2 *β* = 0.722, *p* < 0.001; H3 *β* = 0.830, *p* < 0.001; H4 *β* = 0.838, *p* < 0.001). In addition, social presence is significantly related to price (H5 *β* = 0.654, *p* < 0.001), familiarity (H6 *β* = 0.674, *p* < 0.001), and accessibility (H7 β = 0.634, p < 0.001), supporting hypothesis 5–7. The results revealed that trust is a significant predictor of satisfaction, supporting Hypothesis 10 (H10 *β* = 0.312, *p* < 0.001). Then, hypothesis 13 (H13 *β* = 0.383, *p* < 0.001) revealed that accessibility is significantly associated with satisfaction and hypothesis 14 (H14 *β* = 0.941, *p* < 0.001) revealed satisfaction is positively related to continuance intention. Table 2 presents a summary of SEM results.

**Table 2:** The structural model evaluation.

Proposed paths		*β*	*t*-values
H1	Social interaction → Social presence	0.904	11.596**
H2	Social presence → Sustainability	0.722	10.657**
H3	Social presence → Community belonging	0.830	11.121**
H4	Social presence → Trust	0.838	10.569**
H5	Social presence → Price	0.654	9.813**
H6	Social presence → Familiarity	0.674	9.922**
H7	Social presence → Accessibility	0.634	9.543**
H8	Sustainability → Satisfaction	−0.006	−0.138
H9	Community belonging → Satisfaction	0.140	2.297
H10	Trust → Satisfaction	0.312	4.815**
H11	Price → Satisfaction	0.106	2.344*
H12	Familiarity → Satisfaction	0.077	1.021
H13	Accessibility → Satisfaction	0.419	5.597**
H14	Satisfaction → Continuance intention	0.917	16.136**
Total variance explained: *R*^2^_SP_ = 0.817 *R*^2^_SUS_ = 0.702 *R*^2^_CB_ = 0.402 *R*^2^_TR_ = 0.427 *R*^2^_P_ = 0.454 *R*^2^_FAM_ = 0.689 *R*^2^_ACC_ = 0.521 *R*^2^_SATS_ = 0.754 *R*^2^_CI_ = 0.842		*Goodness-of-fit statistics for the baseline model:* *χ*^2^ = 1356.893, df = 608, *χ*^2^/df = 2.232, *p* < 0.01, IFI = 0.928, TLI = 0.921, CFI = 0.928, RMSEA = 0.054.	

*SI* social interaction, *SP* social presence, *SUS* sustainability, *CB* community belonging, *TR* trust, *P* price, *FAM* familiarity, *ACC* accessibility, *SATS* satisfaction, *PR* performance risk, *SR* social risk, *RCP* reciprocity, *CI* continuance intention.

*p <0.05 **p <0.01

### Evaluation of indirect and total effect

Indirect impacts were assessed to see the mediation effects among the study variables in the last stage of data analysis. Social interaction showed a significant indirect effect on most of its indirect relationships. Indirect effect in the relationships between social interaction–social presence–emotional values were significant (*β* = 0.652, *p* < 0.01 on sustainability, *β* = 0.750, *p* < 0.01 on community belonging, and *β* = 0.757, *p* < 0.01 on trust) as well as social interaction–social presence–functional values (*β* = 0.591, *p* < 0.01 on price, *β* = 0.643, *p* < 0.01 on familiarity, and *β* = 0.609, *p* < 0.01 on accessibility). The relationship between social presence–sustainability–satisfaction (*β* = 0.573, *p* < 0.01), social presence–trust–satisfaction (*β* = 0.261, *p* < 0.01) and social presence–accessibility–satisfaction produced a significant indirect effect (*β* = 0.266, *p* < 0.01). Similarly, trust–satisfaction–continuance intention (*β* = 0.286, *p* < 0.01) and accessibility–satisfaction–continuance intention (*β* = 0.385, *p* < 0.01) demonstrated significant indirect effects. Likewise, social interaction–social presence–trust–satisfaction and social interaction–social presence–trust–satisfaction–continuance intention showed a significant indirect effect (*β* = 0.757, *p* < 0.01). Social interaction–social presence–accessibility–satisfaction and social interaction–social presence–accessibility–satisfaction–continuance intention also showed significant indirect effects as well (*β* = 0.609, *p* < 0.01). Social presence–trust-satisfaction–continuance intention also demonstrated a significant indirect effect (*β* = 0.261, *p* < 0.01 on continuance intention). Lastly, significant indirect effects were visible in a relationship within social presence–accessibility–satisfaction–continuance intention (*β* = 0.266, *p* < 0.01 on continuance intention).

Subsequently, the total effect on continuance intention was examined. Total effect on continuance intention is as follows (*β* SI = 0.630**, *β* SP = 0.697**, *β* SUS = −0.006, *β* CB = 0.129, *β* TR = 0.286**, *β* *P* = 0.097, *β* FAM = 0.071, *β* ACC = 0.385**, *β* SATS = 0.917**). A result of indirect effect and total effect examination is presented in Table 3.

**Table 3:** Indirect and total effect assessment.

Indirect path	*β*(*t*-values)	95%CI Lower	95%CI Upper
SI → SP → SUS	0.652**	0.660	0.902
SI → SP → CB	0.750**	0.642	0.872
SI → SP → TR	0.757**	0.696	0.941
SI → SP → P	0.591**	0.526	0.730
SI → SP → FAM	0.643**	0.547	0.747
SI → SP → ACC	0.609**	0.503	0.698
SP → SUS → *SATS*	0.573**	0.485	0.700
SP → CB → SATS	0.116	0.007	0.246
SP → TR → SATS	0.261**	0.169	0.446
SP → P → SATS	0.069	0.003	0.170
SP → FAM → SATS	0.052*	−0.064	0.178
SP → ACC → SATS	0.266**	0.187	0.473
SUS → SATS → CI	−0.006	−0.069	0.061
CB → SATS → CI	0.129	0.007	0.227
TR → SATS → CI	0.286**	0.152	0.389
P → SATS → CI	0.097	0.004	0.186
FAM → SATS → CI	0.071	−0.076	0.205
ACC → SATS → CI	0.385**	0.233	0.532
SI → SP → SUS → SATS	0.652	−0.054	0.046
SI → SP → SUS → SATS → CI	0.652	−0.054	0.047
SI → SP → CB → SATS	0.750	0.006	0.169
SI → SP → CB → SATS → CI	0.750	0.005	0.171
SI → SP → TR → SATS	0.757**	0.119	0.309
SI → SP → TR → SATS → CI	0.757**	0.120	0.321
SI → SP → P → SATS	0.591	0.003	0.116
SI → SP → P → SATS → CI	0.591	0.003	0.115
SI → SP → FAM → SATS	0.643	0.012	0.147
SI → SP → FAM → SATS → CI	0.643	0.012	0.155
SI → SP → ACC → SATS	0.609**	0.138	0.300
SI → SP → ACC → SATS → CI	0.609**	0.145	0.307
SP → SUS → SATS → CI	−0.005	−0.079	0.069
SP → CB → SATS → CI	0.116	0.008	0.254
SP → TR → SATS → CI	0.261**	0.173	0.461
SP → P → SATS → CI	0.069	0.004	0.171
SP → FAM → SATS → CI	0.052	−0.064	0.018
SP → ACC → SATS → CI	0.266**	0.187	0.473
Total impact on CI: *β*_SI_ = 0.630** *β*_SP_ = 0.697** *β*_SUS_ = −0.006 *β*_CB_ = 0.129	*β*_TR_ = 0.286** *β*_P_ = 0.097 *β*_FAM_ = 0.071 *β*_ACC_ = 0.385** *β*_SATS_ = 0.917**	*Goodness-of-fit statistics for the baseline model:* *χ*^2^ = 1356.893, df = 608, *χ*^2^/df = 2.232, *p* < 0.01, IFI = 0.928, TLI = 0.921, CFI = 0.928, RMSEA = 0.054.	

*SI* social interaction, *SP* social presence, *SUS* sustainability, *CB* community belonging, *TR* trust, *P* price, *FAM* familiarity, *ACC* accessibility, *SATS* satisfaction, *PR* performance risk, *SR* social risk, *RCP* reciprocity, *CI* continuance intention.

*p < 0.05; **p < 0.01.

### Moderating effect by comparing chi-square difference

The multi-group invariance analyses were performed to examine the moderating effect of performance risk, social risk, and reciprocity. A chi-square test was also performed to test the invariance between the baseline model (freely estimated) and the nested model (fully constrained).

The goodness-of-fit statistics of the baseline model of the performance risk (*χ*^2^ = 2298.945, df = 1216, *p* < 0.01, *χ*^2^/df = 1.891, TLI = 0.884, IFI = 0.896, CFI = 0.894, RMSEA = 0.046) fulfilled the acceptable threshold. The chi-square difference test between the baseline model and the nested model revealed that a path between satisfaction and continuance intention is not moderated by performance risk (Δ*χ*^2^(1) = 2.587, *p* > 0.05).

The baseline model of the social risk’s goodness-of-fit statistics (*χ*^2^ = 2367.835, df = 1216, *p* < 0.01, *χ*^2^/df = 1.947, TLI = 0.877, IFI = 0.889, CFI = 0.888, RMSEA = 0.048) also satisfied the acceptable threshold. The chi-square difference test between the baseline model and nested model showed that only the path between satisfaction and continuance intention is not moderated by social risk (Δ*χ*^2^(1) = 1.954, *p* > 0.05).

The goodness-of-fit statistics of the baseline model of the reciprocity (*χ*^2^ = 2267.495, df = 1216, *p* < 0.01, *χ*^2^/df = 1.865, TLI = 0.863, IFI = 0.877, CFI = 0.875, RMSEA = 0.046) met the acceptable threshold. The chi-square difference test between the baseline model and nested model confirmed that the path between satisfaction and continuance intention is significantly moderated by reciprocity (Δ*χ*^2^(1) = 5.079, *p* > 0.05). Table 4 displays a full summary of the invariance test results.

**Table 4:** Assessment of multigroup moderating effect.

Moderation test for performance risk^a^						
Paths	Low-performance risk		High-performance risk		Baseline model	Nested model
	*β*	*t*-values	*β*	*t*-values		
SATS → CI	0.860	12.367**	0.915	8.676**	*χ*^2^(1216) = 2298.945	*χ*^2^(1217) = 2301.532
*Chi-square difference test*						
Δ*χ*^2^(1) = 2.587, *p* > 0.05 (H15i: Not supported)						
*Moderation test for social risk*^b^						
**Paths**	**Low social risk**		**High social risk**		**Baseline model**	**Nested model**
	***β***	***t*-values**	***β***	***t*-values**		
SATS → CI	1.098	11.152**	0.921	11.331**	*χ*^2^(1216) = 2367.835	*χ*^2^(1217) = 2369.789
*Chi-square difference test:*						
Δ*χ*^2^(1) = 1.954, *p* > 0.05 (H16i: Not supported)						
*Moderation test for reciprocity*^c^						
**Paths**	**Low reciprocity**		**High reciprocity**		**Baseline model**	**Nested model**
	***β***	***t*-values**	***β***	***t*-values**		
SATS → CI	1.129	9.699**	0.792	8.259**	*χ*^2^(1216) = 2267.495	*χ*^2^(1217) = 2272.574
*Chi-square difference test*						
Δ*χ*^2^(1) = 5.079, *p* > 0.05 (H17i: Supported)						

^a^*Goodness-of fit indices of the baseline model:* *χ*^2^ = 2298.945, df = 1216, *p* < 0.01, *χ*^2^/df = 1.891, TLI = 0.884, IFI = 0.896, CFI = 0.894, RMSEA = 0.046.

*SATS* satisfaction, *CI* continuance intention.

**p < 0.01.

^b^*Goodness-of fit indices of the baseline model:* *χ*^2^ = 2367.835, df = 1216, *p* < 0.01, *χ*^2^/df = 1.947, TLI = 0.877, IFI = 0.889, CFI = 0.888, RMSEA = 0.048.

*SATS* satisfaction, *CI* continuance Intention.

**p < 0.01.

^c^*Goodness-of fit indices of the baseline model:* *χ*^2^ = 2267.495, df = 1216, *p* < 0.01, *χ*^2^/df = 1.865, TLI = 0.863, IFI = 0.877, CFI = 0.875, RMSEA = 0.046.

SATS satisfaction, *CI* continuance intention.

**p < 0.01.

## Discussion and conclusion

The sharing economy is still growing in the hospitality industry and is expected to continue to grow. This study identifies social factors of the sharing economy in the hospitality industry which attracted not much attention in extant literature. It also confirms the attributes of intrinsic and extrinsic values of the sharing economy in the hospitality industry. These attributes were studied generally not in the hospitality context (Möhlmann, 2015). In building continuance intention, this study validates the role of customer satisfaction as a mediator. This study also identifies the moderating role of perceived risks and reciprocity in the hospitality businesses participating in the sharing economy. Thus, the study delivers several theoretical implications to hospitality research and provides practical implications to hospitality businesses.

### Implications

First, this research has confirmed that social interaction and social presence are two fundamental factors that affect how individuals perceive the hospitality businesses participating in the sharing economy, signaling the hospitality business operators to be concerned about social interaction between businesses and customers and/or customers. In addition, the hospitality business operators should acknowledge which of the emotional and functional values were significantly associated with a social presence, affecting customer satisfaction. For example, hospitality business operators should try to increase individuals’ trust during an interaction with each other when using the sharing economy services as social presence affects trust. A detailed description of the hospitality businesses’ services and a prompt response to customers’ questions and/or feedback are likely to increase an individual’s trust in the sharing economy services. A “trust resides in a range of individual consumer characteristics, website and firm feature and the interactions between a firm and the consumer” (Chen and Dhillon, 2003). Corresponding to the data results, the hospitality business operators should also consider ways to increase the accessibility of their business services by increasing the number of properties of the hospitality businesses. Likewise, hospitality business operators should also try to increase the legibility of their business platforms. This is because legibility, known as ease of navigation, increases customers’ satisfaction (Yeh and Li, 2014). Hence, hospitality business operators shall find ways to increase accessibility both offline and online. Also, they should also consider ways to increase reciprocity in sharing economy as it increases continuance intention.

This study has made a huge contribution to current knowledge of the sharing economy in the hospitality industry. The research outcome not only refines the impact of trust in the sharing economy but also investigates other values of the sharing economy. Moreover, the research sample improves Möhlmann’s finding that satisfaction is positively correlated with the likelihood of using sharing option again (Möhlmann, 2015) by testing customers’ continuance intention, Furthermore, the research examines the moderating effects of perceived performance risks, social risks, and reciprocity on satisfaction and continuance intention to add a value to the extant research analysis. Even though there are extant studies that have investigated the determinants of the sharing economy, factors like perceived performance risk, social risk, and reciprocity were not often investigated as moderators and remain unearthed. For this reason, this research has enhanced the current knowledge on moderating effects of perceived risks and reciprocity in customers’ behavior intention in the sharing economy context.

### Limitations and suggestions for future research

Even though this research meaningfully uncovers the hospitality businesses participating in the sharing economy, there are several limitations to this research. First, the samples were only collected in Korea, and the hospitality businesses were selected based on the researchers’ recognition of the businesses’ presence in society. Thus, it is important to acknowledge that some of these hospitality businesses have no presence overseas.

Second, although data samples were collected based on three different hospitality business sectors that are participating in the sharing economy, the data samples were analyzed as one big data sample. Therefore, the present research should inspire future research to investigate the data samples from each business sector. Lastly, the last construct of the study model is continuance intention. Instead of studying the intention of the customers, future research shall examine the actual continuances or revisits.

## Data Availability

The datasets generated during and/or analyzed during the current study are available from the corresponding author on reasonable request.
